# Scattering Theory of Graphene Grain Boundaries

**DOI:** 10.3390/ma11091660

**Published:** 2018-09-08

**Authors:** Francesco Romeo, Antonio Di Bartolomeo

**Affiliations:** Dipartimento di Fisica “E. R. Caianiello”, Università di Salerno, I-84084 Fisciano, Italy; adibartolomeo@unisa.it

**Keywords:** graphene grain boundaries, scattering matrix theory, Dirac Hamiltonian

## Abstract

The implementation of graphene-based electronics requires fabrication processes that are able to cover large device areas, since the exfoliation method is not compatible with industrial applications. The chemical vapor deposition of large-area graphene represents a suitable solution; however, it has an important drawback of producing polycrystalline graphene with the formation of grain boundaries, which are responsible for the limitation of the device’s performance. With these motivations, we formulate a theoretical model of a single-layer graphene grain boundary by generalizing the graphene Dirac Hamiltonian model. The model only includes the long-wavelength regime of the charge carrier transport, which provides the main contribution to the device conductance. Using symmetry-based arguments deduced from the current conservation law, we derive unconventional boundary conditions characterizing the grain boundary physics and analyze their implications on the transport properties of the system. Angle resolved quantities, such as the transmission probability, are studied within the scattering matrix approach. The conditions for the existence of preferential transmission directions are studied in relation with the grain boundary properties. The proposed theory provides a phenomenological model to study grain boundary physics within the scattering approach, and represents *per se* an important enrichment of the scattering theory of polycrystalline graphene. Moreover, the outcomes of the theory can contribute to understanding and limiting the detrimental effects of graphene grain boundaries, while also providing a benchmark for more elaborate techniques.

## 1. Introduction

Graphene (G) is a two-dimensional honeycomb lattice that is constituted by carbon atoms. Its reciprocal lattice determines a hexagonal Brillouin zone that has six corners (K/K’ points) where the low-energy part of the band’s structure is well described by a linear energy–momentum dispersion relation, defining the so-called Dirac cone. The existence of Dirac cones in the graphene band’s structure can be understood by using a tight-binding model. Consequently, the electron motion in the graphene lattice follows the Dirac equation [1], with the latter being the manifestation of an emergent ultra-relativistic behavior in a many-body system that was initially described by the Schrödinger equation. Due to its unique band structure, in the past few years, graphene has attracted much attention and subsequently its intriguing transport properties, such as Klein tunneling [2], Zitterbewegung effect [3], antilocalization [4], anomalous quantum Hall effect [5], and Veselago focusing effect [6], have been suggested and, in some cases, experimentally proven. Apart from its theoretical interest, graphene is a two-dimensional chemical homogeneous system that is characterized by very high electrical mobility [7] and extraordinary mechanical properties [8], making it appealing in nanoelectronics and for flexible electronics implementations [9]. Due to its single-atom-thick nature, graphene appears to be the ideal candidate to study field effect devices [10]. However, common problems with graphene-based field effect transistors (FET) are: device performances are usually limited by the electrode–graphene contact resistance [11,12,13,14]; the position of the Dirac point is strongly affected by a random doping induced in the fabrication process; a low on–off currents ratio in G-FET compared to the commercial silicon-based FET, due to the lack of a bandgap; graphene channel contamination [15,16] occurs quite easily due to chemical impurities, ambient conditions (e.g., humidity), etc.; Dirac point modulation along the graphene channel due to the chemical doping induced by the electrodes and controlled by the difference of the work functions at the metal–graphene interface.

Moreover, the implementation of graphene-based electronics requires the chemical vapor deposition technique, which is able to cover large device areas and is compatible with industrial fabrication processes. However, an important drawback of this technique is represented by the polycrystalline nature of the samples. Polycrystalline graphene presents grain boundaries [17,18,19], which are responsible for the limitation of the device’s performance. For this reason, the morphology [20,21] and the electrical properties [22,23] of grain boundaries in polycrystalline graphene have received great attention in the last decade. In particular, it has been shown that one-dimensional defects in graphene accumulate electrostatic charge via a self-doping mechanism [24]. Self-doping takes place because one-dimensional defects in the *sp*^2^ carbon structure accommodate localized states, acting as trapping centers for the charge carriers. The effects of charged line defects in graphene have been studied in the literature and are recognized as an important factor for mobility degradation in chemically vapor-deposited graphene [25,26].

From a theoretical point of view, linear defects in graphene have been studied by J.N.B. Rodrigues and coworkers in Refs. [27,28,29,30], where specific boundary conditions have been derived from the tight-binding model of the extended defect. The mentioned approach provides a continuous model to study transport properties in the presence of selected linear defects, whose atomic arrangement determines the boundary conditions of the scattering problem.

In this work, we provide an alternative method and develop a continuous model of polycrystalline graphene in which general boundary conditions are derived without specifying the microscopic structure of the linear defect.

With these motivations, we formulate a theoretical model of the graphene grain boundary by generalizing the graphene Dirac Hamiltonian model. A continuous approach is used that is justified within the long wavelength limit assumed throughout this work. Using symmetry-based arguments, we derive unconventional boundary conditions characterizing the grain boundary physics, and analyze their implications on the transport properties of the system. The scattering matrix approach [31] is used to derive the conduction properties of the grain boundary interface, and angle-resolved quantities, such as the transmission probability, are presented. The conditions for the existence of preferential transmission directions are studied in connection with the grain boundary properties.

The work is organized as follows. In Section 2, we derive the Dirac Hamiltonian within a rotated reference frame, current density conservation, and boundary conditions at a grain boundary. The matching matrix method is introduced and adapted to the scattering problem at the grain boundary. In Section 3, we formulate a grain boundary Hamiltonian model with the position-dependent rotation angle θ(x). The model is studied by using space-dependent unitary transformation, which allows the use of conventional boundary conditions in the scattering problem. In Section 4, the results of the scattering theory are reported. Conclusions are given in Section 5. Appendix A is included to discuss K point displacement in momentum space induced by off-diagonal potentials in sublattice representation.

## 2. Dirac Hamiltonian within a Rotated Reference Frame, Current Density Conservation, and Boundary Conditions at a Grain Boundary

In this section, we provide a first approach to the grain boundary problem in monolayer graphene, while studying the grain boundaries in multilayer graphene is beyond the purposes of this work. We deal with a theory of spinless particles described by the continuous Dirac Hamiltonian, and consider a single valley degree of freedom. Neglecting the electron spin is appropriate in the absence of magnetic fields or magnetized regions, which is the case considered in this work. On the other hand, describing graphene electrons adopting a single-valley perspective requires an appropriate justification. Indeed, in principle, the grain boundary region can be the source of valley-flipping scattering events. The existence of such kind of phenomena has inspired the emerging field of *valleytronics* [32], aiming at manipulating the valley degree of freedom as it is done with the spin in *spintronics*. However, valleytronics manipulations require engineered scattering centers, which are quite challenging to implement using current technologies. In view of this circumstance, we can argue that valley-mixing scattering events are quite rare in spontaneously formed grain boundaries. Moreover, to a good approximation, electrons that have different valley degrees of freedom can be described as two independent quantum fluids. The latter argument justifies the single-valley treatment that is adopted. Generalization of the ideas exposed hereafter to spinful particles having two-valley degrees of freedom is in principle immediate.

### 2.1. Dirac Hamiltonian within a Rotated Reference Frame

In this subsection, we describe the continuous Dirac Hamiltonian of a single graphene sheet within a rotated reference frame. The continuous approach considered in this work is appropriate for describing the system conduction properties (e.g., the differential conductance) when the electron wavelength, which is associated with the particle momentum, is much longer than the interatomic distance of the honeycomb lattice. While the latter assumption is clearly invalidated in graphene nanoribbons devices, it is appropriate for a large variety of graphene-based devices where micrometric graphene sheets are employed as conduction channels.

**Figure 1:**
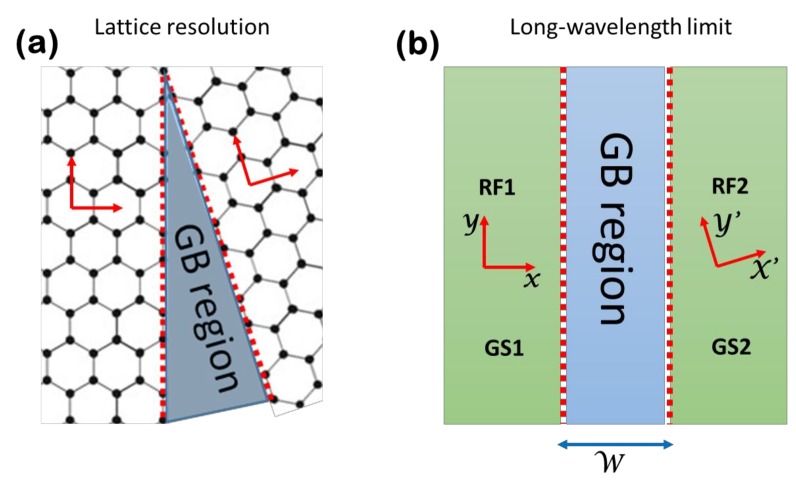
(**a**) Schematic of the region close to a grain boundary (GB region). Apart from the grain boundary region, where lattice distortions and vacancies affect the honeycomb lattice structure of graphene, the bulk of the system is described by a regular atomic arrangement in which the lattice orientation is subject to a rotation of a definite angle going from the left to the right side of the junction. (**b**) The long wavelength limit of the grain boundary junction depicted in panel (**a**). The graphene sheets 1 and 2, namely GS1 and GS2, represent the two sides of the junction, while the GB region is represented by a region of negligible extension *W*, whose effects on the scattering properties of the junction are captured by appropriate boundary conditions. The boundary conditions are a direct manifestation of the presence of a grain boundary effective potential U(x). The presence of the GB imposes a lattice rotation of the GS2 compared to the GS1. This effect is easily described using distinct local reference frames, RF1 and RF2, in describing the two sides of the system. Translational invariance along the y-direction of the RF1 is assumed.

Let us describe the Dirac Hamiltonian of the graphene sheet GS2 in Figure 1, in terms of the reference frame RF1 of the graphene sheet GS1. The Hamiltonian of GS2 written in terms of reference frame RF2 takes the usual form:

(1) $$H_{\xi }=v_{F}\left(\begin{array}{cc} 0 & \xi {\hat{p}}_{x^{\prime }}-i{\hat{p}}_{y^{\prime }}\\ \xi {\hat{p}}_{x^{\prime }}+i{\hat{p}}_{y^{\prime }} & 0 \end{array}\right),$$

where ξ=±1 represents the valley quantum number and $v_{F}$ is the Fermi velocity, while ${\hat{p}}_{x^{\prime }}=-i\hbar \partial_{x^{\prime }}$ and ${\hat{p}}_{y^{\prime }}=-i\hbar \partial_{y^{\prime }}$ are the quantum mechanical operators associated with the linear momentum components of quasiparticles. The matrix structure of the Hamiltonian originates from the presence of two atoms inside the unit cell of the graphene Bravais lattice. Accordingly, the wave function $\Psi \left(x^{\prime },y^{\prime }\right)={\left(\Psi_{A},\Psi_{B}\right)}^{T}$ describing the quantum state of the charge carriers is a two-component spinor whose components are related to the probability of finding the particle on atom A or B of the unit cell. For the reasons explained above, we neglect the valley quantum number and set ξ=1. The correspondence between the wave functions expressed in RF1 or RF2 is established, observing that the α-component of the wave function written in RF1, i.e., $\Phi_{\alpha }\left(x,y\right)$, is related to the homologous component in RF2 by the equation $\Phi_{\alpha }\left(x,y\right)=\Psi_{\alpha }\left(x^{\prime }\left(x,y\right),y^{\prime }\left(x,y\right)\right)$, with α∈{A,B}. The relation between the distinct reference frames is simply given by a two-dimensional rotation:

(2) $$\left(\begin{array}{c} x^{\prime }\\ y^{\prime } \end{array}\right)=\left(\begin{array}{cc} \cos\theta & \sin\theta \\ -\sin\theta & \cos\theta \end{array}\right)\left(\begin{array}{c} x\\ y \end{array}\right),$$

with θ being an appropriate rotation angle. From the above observations, one obtains useful relations linking the partial derivatives of the wave functions in the form:

(3) $$\left(\begin{array}{c} \partial_{x}\Phi_{\alpha }\left(x,y\right)\\ \partial_{y}\Phi_{\alpha }\left(x,y\right) \end{array}\right)=\left(\begin{array}{cc} \cos\theta & -\sin\theta \\ \sin\theta & \cos\theta \end{array}\right)\left(\begin{array}{c} \partial_{x^{\prime }}\Psi_{\alpha }\left(x^{\prime },y^{\prime }\right)\\ \partial_{y^{\prime }}\Psi_{\alpha }\left(x^{\prime },y^{\prime }\right) \end{array}\right).$$

Using Equation (3) in evaluating the quantity $H_{+}\Psi \left(x^{\prime },y^{\prime }\right)$, one easily gets the equality:

$$H_{+}\Psi \left(x^{\prime },y^{\prime }\right)=H_{\theta }\Phi \left(x,y\right),$$

where:

(4) $$H_{\theta }=v_{F}\left(\begin{array}{cc} 0 & e^{i\theta }\left({\hat{p}}_{x}-i{\hat{p}}_{y}\right)\\ e^{-i\theta }\left({\hat{p}}_{x}+i{\hat{p}}_{y}\right) & 0 \end{array}\right)$$

represents the Hamiltonian of GS2 written in terms of RF1. Solving the eigenvalues problem $H_{\theta }\psi =E\;\psi$, with the usual planewave ansatz $\psi ={\left(\alpha ,\beta \right)}^{T}e^{i\left(k_{x}x\;+\;k_{y}y\right)}$, one finds eigenstates:

(5) $$\psi_{\nu }=\frac{1}{\sqrt{2}}\left(\begin{array}{c} 1\\ \nu e^{i\left(\varphi -\theta \right)} \end{array}\right)e^{i\vec{k}\cdot \vec{r}},$$

with associated eigenvalues $E_{\nu }=\nu \hbar v_{F}\left|k\right|$. Here, ν=±1 represents the band index (ν=1 for conduction band and ν=−1 for valence band), $\vec{k}=\left|k\right|\left(\cos\left(\varphi \right),\sin\left(\varphi \right)\right)$ is the particles wavevector, and $\vec{r}=\left(x,y\right)$ is the coordinates vector. The above results show that the physical properties of the system, e.g., the energy spectrum, are reference-frame independent and are clearly reminiscent of the rotational invariance of a bulk (monocrystalline) graphene sheet. Up to now, we have limited our attention to the problem of a single graphene sheet and its description within a rotated reference frame. We have found that the rotated Hamiltonian depends on a phase factor $e^{\pm i\theta }$, which is not observable. Hereafter, we study the junction depicted in Figure 1, where graphene sheets with different reticular orientations are connected by means of a grain boundary region. Setting the RF1 as a global reference frame, the Hamiltonian of the entire system $H_{gb}$ takes the piecewise form:

(6) $$H_{gb}=\{\begin{array}{cc} H_{\theta \to 0} & x<0\\ H_{\theta } & x>0 \end{array}$$

The line x=0 defines the grain boundary region in the limit W→0, which is an appropriate approximation when the grain boundary extension W along the x-direction is negligible. This requirement is always met by real grain boundaries, which are defected regions where W covers at least a few lattice sites. The latter observation suggests that the scattering properties of the grain boundary region are adequately captured by appropriate boundary conditions within the framework of a continuous model. Such boundary conditions are non-standard, and are the object of the subsequent analyses. Indeed, when the grain boundary problem formalized so far is treated using ordinary boundary conditions, a violation of the current conservation is found, which is a clear indication that modified boundary conditions are required. Thus, the solution of the problem first requires the derivation of the current density conservation law.

### 2.2. Current Density Conservation

Before treating the grain boundary problem, let us consider the current density conservation law originated by the rotated Hamiltonian (4). A continuity equation in the form $\partial_{t}\rho +\vec{\nabla }\cdot \vec{J}=0$ is obtained by taking the time derivative of the charge density $\rho =\Psi^{+}\Psi ={\left|\Psi_{A}\right|}^{2}+{\left|\Psi_{B}\right|}^{2}$ (the electric charge is omitted) and using in the computation the rotated Dirac equation in the form $\partial_{t}\Psi ={\left(i\hbar \right)}^{-1}H_{\theta }\Psi$. After straightforward computation, the current density components, namely $J_{x}=\Psi^{+}{\hat{J}}_{x}\Psi$ and $J_{y}=\Psi^{+}{\hat{J}}_{y}\Psi$, are easily recognized. Here, the first quantized current density operators take the following form:

(7) $${\hat{J}}_{x}=v_{F}\left(\begin{array}{cc} 0 & e^{i\theta }\\ e^{-i\theta } & 0 \end{array}\right)$$

(8) $${\hat{J}}_{y}=v_{F}\left(\begin{array}{cc} 0 & -ie^{i\theta }\\ ie^{-i\theta } & 0 \end{array}\right),$$

whose structure explicitly depends on the rotation angle θ. It is worth mentioning here that the expressions for the θ=0 case coincide with the usual relations ${\hat{J}}_{x}=v_{F}{\hat{\sigma }}_{x}$ and ${\hat{J}}_{y}=v_{F}{\hat{\sigma }}_{y}$, where ${\hat{\sigma }}_{x}$ and ${\hat{\sigma }}_{y}$ represent ordinary Pauli matrices. In the following discussion, we denote the quantities in Equations (7) and (8) as ${\hat{J}}_{x,y}^{\theta }$ in order to stress the dependence on the rotation angle θ, while notation ${\hat{J}}_{x,y}^{0}$ will be adopted to indicate the same quantities when θ=0.

### 2.3. The Mathematical Problem of Boundary Conditions at a Grain Boundary

We are now ready to treat the problem of a grain boundary. This situation is schematized using the Hamiltonian model given in Equation (6). Accordingly, the current density operator does not admit a global definition, while the following piecewise definition:

(9) $${\hat{J}}_{x,y}\left(x\right)=\{\begin{array}{cc} {\hat{J}}_{x,y}^{0} & x<0\\ {\hat{J}}_{x,y}^{\theta } & x>0 \end{array}$$

is required. The form of Equation (9) fully explains the ultimate reasons of the failure of usual boundary conditions in describing the scattering problem defined by the Hamiltonian in Equation (6). Hereafter, we provide a careful explanation of this point. First of all, we explicitly observe that under translational invariance along the y-axis, the y-component of the current density, namely $J_{y}=\Psi^{+}{\hat{J}}_{y}\Psi$, does not depend on the coordinate y, and thus the quantity $\partial_{y}J_{y}=0$ does not play any role in the continuity equation. Therefore, current density conservation entirely depends on the conservation of the x-component of the current density. The current density conservation problem is related to the invariance of the current density operator under appropriate transformations. This point can be easily understood by observing that, within the transfer matrix formalism, boundary conditions for the spinorial wave function at the x=0 interface can be written as $\Psi \left(0^{+}\right)=ℳ\Psi \left(0^{-}\right)$, with ℳ being a 2×2 matching matrix. Here, we have introduced the notation $0^{+}$ and $0^{-}$, meaning a spatial position close to x=0 and belonging to the right or left side of the junction, respectively. Within this formalism, the current density on the right (left) side of the interface, namely $J_{R}$ ($J_{L}$), takes the form of $J_{R}=\Psi^{+}\left(0^{+}\right){\hat{J}}_{x}\left(0^{+}\right)\Psi \left(0^{+}\right)$ ($J_{L}=\Psi^{+}\left(0^{-}\right){\hat{J}}_{x}\left(0^{-}\right)\Psi \left(0^{-}\right)$), while current density conservation requires the condition $J_{R}=J_{L}$. Observing that $J_{R}=\Psi^{+}\left(0^{-}\right){ℳ}^{+}{\hat{J}}_{x}\left(0^{+}\right)ℳ\Psi \left(0^{-}\right)$ and using the continuity of the current density at the interface, we get the important relation ${ℳ}^{+}{\hat{J}}_{x}\left(0^{+}\right)ℳ={\hat{J}}_{x}\left(0^{-}\right)$. Here, we explicitly notice that the current density operator in the absence of lattice mismatching at the interface (i.e., θ=0) is globally defined so that ${\hat{J}}_{x}\left(0^{+}\right)={\hat{J}}_{x}\left(0^{-}\right)$, and the usual boundary conditions are recovered. This is not the case for the problem under study where ${\hat{J}}_{x}\left(0^{+}\right)\neq {\hat{J}}_{x}\left(0^{-}\right)$, as evident by direct inspection of Equation (9). According to the above arguments, proper boundary conditions for the scattering problem at the grain boundary interface are assigned once an opportune matching matrix ℳ has been identified. Based on the current density conservation law, a matching matrix ℳ has to respect the following matrix equation:

(10) $${ℳ}^{+}{\hat{J}}_{x}^{\theta }ℳ=v_{F}{\hat{\sigma }}_{x}\Rightarrow {ℳ}^{+}\left(\begin{array}{cc} 0 & e^{i\theta }\\ e^{-i\theta } & 0 \end{array}\right)ℳ={\hat{\sigma }}_{x}$$

for arbitrary choices of the rotation angle, θ. The matching matrix ℳ is completely determined by the properties of the grain boundary local potential U(x) to be added to the Dirac Hamiltonian (6). Such potential is a 2×2 Hermitian operator, which is different from zero only inside the grain boundary region (the GB region in Figure 1b), while its specific form depends on the realization of the interface between grains at the atomic level. The latter information is clearly not available within the framework of a long wavelength (continuous) model, and thus, U(x) has to be meant as a phenomenological potential that is able to reproduce the transport properties of the interface. A useful simplification that will be used in the following discussion parameterizes the interface potential as U(x)=ℬδ(x) with δ(x) being the Dirac delta function and ℬ as a 2×2 Hermitian operator. Once the structure of U(x)=ℬδ(x) is known, the matching matrix ℳ can be determined as described in Section 2.5.

An alternative and interesting approach is completely based on the algebraic properties of the matching matrix ℳ. The algebraic method allows the identification of all of the possible matching matrices in the absence of any information on the scattering potential U(x). The generality of this approach, which will be presented in Section 2.4, is quite appealing in describing real systems where precise information on the interface are not available.

### 2.4. Algebraic Classification of the Matching Matrix ℳ for the Grain Boundary Problem

In this subsection, we study the general problem of a grain boundary formed between two graphene sheets whose reticular axes are rotated by the angles γ and θ, respectively. Under this general condition, the grain boundary Dirac Hamiltonian takes the following piecewise form:

(11) $$H_{gb}=\{\begin{array}{cc} H_{\gamma } & x<0\\ H_{\theta } & x>0 \end{array}.$$

Moreover, we assume that an unknown grain boundary potential U(x) is present at the interface located at x=0. Our purpose is deriving the general structure of all of the possible matching matrices *ℳ* and disregarding the unknown properties of the grain boundary potential. First of all, we do observe that current density conservation takes the general form ${ℳ}^{+}{\hat{J}}_{x}^{\theta }ℳ={\hat{J}}_{x}^{\gamma }$, which is insensitive to the transformation $ℳ\to e^{i\varphi }ℳ$, with φ being an arbitrary phase factor. The latter property will be taken into account in the following presentation by omitting the arbitrary phase factor.

In order to follow our program, we consider two relevant classes of matching matrices: (*i*) matching matrices belonging to the special unitary group SU(2) constituted by 2×2 unitary matrices with a determinant equal to 1; (*ii*) matching matrices belonging to the special linear group SL(2,C) constituted by 2×2 matrices with a determinant equal to 1 defined over the complex number field.

Let us start with the case of ℳ∈SU(2). This class of matching matrices contains the identity matrix $ℳ=1_{2\times 2}$ which is involved in the matching condition $\Psi \left(0^{+}\right)=\Psi \left(0^{-}\right)$ (continuity of the wave function) used, for instance, in describing the Klein tunneling phenomenon through a rectangular electrostatic potential. We solve the matrix equation coming from the current density conservation by substituting the following general parameterization of ℳ∈SU(2):

(12) $$ℳ=\left(\begin{array}{cc} e^{i\left(\alpha +\beta \right)}cos\left(\lambda \right) & e^{i\left(\alpha -\beta \right)}sin\left(\lambda \right)\\ -e^{-i\left(\alpha -\beta \right)}sin\left(\lambda \right) & e^{-i\left(\alpha +\beta \right)}cos\left(\lambda \right) \end{array}\right),$$

where α, β, λ are real parameters to be determined. The solution of the matrix equation allows the identification of the unknown parameters that appear in Equation (12). After straightforward computation, we obtain that a general SU(2) matching matrix takes the form:

(13) $${ℳ}_{1}=\left(\begin{array}{cc} e^{i\left(\frac{\theta -\gamma }{2}\right)}cos\left(\lambda \right) & ie^{i\left(\frac{\theta +\gamma }{2}\right)}sin\left(\lambda \right)\\ ie^{-i\left(\frac{\theta +\gamma }{2}\right)}sin\left(\lambda \right) & e^{-i\left(\frac{\theta -\gamma }{2}\right)}cos\left(\lambda \right) \end{array}\right).$$

As expected, ${ℳ}_{1}$ only depends on the relative angle θ−γ≠0 formed by the crystallographic axes of the graphene sheets forming the two sides of the grain boundary junction. Interestingly, ${ℳ}_{1}$ cannot be equal to the identity matrix within the SU(2) parametrization, and thus the usual boundary condition $\Psi \left(0^{+}\right)=\Psi \left(0^{-}\right)$ is not compatible with the current density conservation in the presence of a grain boundary. In particular, while the off-diagonal contribution in ${ℳ}_{1}$ can be eliminated by setting λ=0, diagonal terms are always different from one when θ−γ≠0, with the latter being the condition of interest in the grain boundary case. Ordinary boundary conditions, namely $\Psi \left(0^{+}\right)=\Psi \left(0^{-}\right),$ are correctly recovered by setting θ−γ=0. The physical meaning of the parameter λ will be clarified in Section 2.5.

A similar procedure can be applied to the case of ℳ∈SL(2,C), where the unitary condition is relaxed compared to the previous case. Under this assumption, we obtain the following matching matrix parameterizations:

(14) $${ℳ}_{2}=\left(\begin{array}{cc} a\;e^{i\left(\frac{\theta -\gamma }{2}\right)} & -ib\;e^{i\left(\frac{\theta +\gamma }{2}\right)}\sigma \\ ic\;e^{-i\left(\frac{\theta +\gamma }{2}\right)} & d\;e^{-i\left(\frac{\theta -\gamma }{2}\right)}\sigma \end{array}\right),$$

with a, b, c, d ≥0, |ad−bc|=1 and σ=sign(ad−bc). An additional matching matrices set, which cannot be obtained from Equation (14) under suitable assumptions, is given by:

(15) $${ℳ}_{3}=\left(\begin{array}{cc} a\;e^{i\left(\frac{\theta -\gamma }{2}\right)} & ib\;e^{i\left(\frac{\theta +\gamma }{2}\right)}\\ \;ic\;e^{-i\left(\frac{\theta +\gamma }{2}\right)} & d\;e^{-i\left(\frac{\theta -\gamma }{2}\right)} \end{array}\right),$$

with a, b, c, d ≥0 and ad+bc=1. Interestingly, the only diagonal matching matrix such that ℳ∈SL(2,C) takes the form ${ℳ}_{diag}=diag\left(a\;e^{i\left(\frac{\theta -\gamma }{2}\right)},a^{-1}\;e^{-i\left(\frac{\theta -\gamma }{2}\right)}\right)$, with *a* > 0. Moreover, matching matrices belonging to SL(2,C) admit a description with three parameters, while SU(2) parameterization requires only one parameter.

The matching matrices ${ℳ}_{1,2,3}$ define a rather general set of physically relevant matching matrices whose structure is fully defined by algebraic properties that are the direct manifestation of the conservation laws of the problem. The matching matrix parameterizations given in Equations (13)–(15) are one of the main results of this work. The physical consequences of the matching matrix structure and its algebraic properties will be discussed later on.

### 2.5. Direct Derivation of the Matching Matrix ℳ from the Scattering Potential U(x)=ℬδ(x)

In this section, we provide a link between the features of the scattering potential U(x) and the parameters a,b,c,d, λ that were introduced in Equations (13)–(15). To follow this program, we set aside the problem of grain boundary for a moment and focus our attention on the scattering problem described by the Hamiltonian $H=H_{\theta \to 0}+ℬ\delta \left(x\right)$, with ${ℬ}^{+}=ℬ$ being an Hermitian operator in the sublattice space. As will be discussed in Section 3, this problem is related to the grain boundary problem by a local unitary transformation, and thus it is not collateral in our discussion. First of all, we do observe that deriving proper boundary conditions for a Dirac delta potential in a Dirac equation requires some care [33]. Indeed, the usual procedure that is followed in the case of the Schrödinger equation for massive particles does not work when the quantum operator associated with the particle kinetic energy is represented by a first-order derivative with respect to the space variable. Under this circumstance, the particle wave function Ψ(x) is not constrained to be continuous in x=0 (i.e., $\Psi \left(0^{+}\right)\neq \Psi \left(0^{-}\right)$), and thus the integral $\int_{-\epsilon }^{\epsilon }\Psi \left(x\right)\;\delta \left(x\right)\;dx$, with ϵ>0, is an ill-defined quantity. In principle, since δ(x)=δ(−x), one may argue that $\int_{-\epsilon }^{\epsilon }\Psi \left(x\right)\;\delta \left(x\right)\;dx=\left[\Psi \left(0^{+}\right)+\Psi \left(0^{-}\right)\right]/2$, but this conclusion is incorrect and leads to contradictions. To avoid these difficulties, we provide an alternative derivation. Let us start with the stationary Dirac equation HΨ(x,y)=EΨ(x,y), with $\Psi \left(x,y\right)=\Psi \left(x\right)e^{i\;k_{y}y}$ due to the translational invariance along the y-direction. Since we are interested in the wave function behavior in close vicinity of x=0, we only retain the diverging potential in the Dirac equation. This procedure leads to the first-order differential equation $\partial_{x}\Psi \left(x\right)=\hat{T}\left(x\right)\Psi \left(x\right)$, with $\hat{T}\left(x\right)=-i{\left(\hbar v_{F}\right)}^{-1}\delta \left(x\right){\hat{\sigma }}_{x}ℬ$, whose formal solution shares similarities with that of a time-dependent Schrodinger problem in which time-ordered operators are involved. Similarly, the formal solution of our problem involves the definition of a space-ordering operator $P_{x}[\ldots ]$ instead of the usual time-ordering operator. In particular, the formal solution can be derived by integration with the following result:

(16) $$\Psi \left(x\right)=\Psi \left(x_{0}\right)+\int_{x_{0}}^{x}\hat{T}\left(y\right)\Psi \left(y\right)\;dy$$

An infinite iteration of Equation (16) leads to the formal solution $\Psi \left(x\right)=P_{x}[exp(\int_{x_{0}}^{x}\hat{T}\left(y\right)dy)]\Psi \left(x_{0}\right)$, where the action of the space-ordering operator is defined as $P_{x}\left[A\left(x_{1}\right)B\left(x_{2}\right)\right]=A\left(x_{1}\right)B\left(x_{2}\right)\theta (x_{1}-x_{2})+B\left(x_{2}\right)A\left(x_{1}\right)\theta (x_{2}-x_{1})$, while $\theta \left(x_{1}-x_{2}\right)$ represents the Heaviside step function. An important observation for our derivation is that when the commutator $\left[A\left(x_{1}\right),B\left(x_{2}\right)\right]$ is a vanishing quantity, space ordering simply gives $P_{x}\left[A\left(x_{1}\right)B\left(x_{2}\right)\right]=A\left(x_{1}\right)B\left(x_{2}\right)$. When the above arguments are applied to our original problem using a limit procedure (i.e., $x_{0}\to -\epsilon$ and x→ϵ, with ϵ→0), we immediately get:

(17) $$\Psi \left(0^{+}\right)=exp\left(-i{\left(\hbar v_{F}\right)}^{-1}{\hat{\sigma }}_{x}ℬ\right)\Psi \left(0^{-}\right)$$

A direct inspection of Equation (17) allows us to identify the matching matrix $ℳ=exp\left(-i{\left(\hbar v_{F}\right)}^{-1}{\hat{\sigma }}_{x}ℬ\right)$ and its dependence on the scattering potential. In the following, we provide relevant examples of matching matrices based on the analysis of Equation (17). We use the general parameterization for the Hermitian matrix ${\left(\hbar v_{F}\right)}^{-1}ℬ$ reported below:

(18) $${\left(\hbar v_{F}\right)}^{-1}ℬ\;=\left(\begin{array}{cc} w_{a} & w_{X}e^{-i\varphi }\\ w_{X}e^{i\varphi } & w_{b} \end{array}\right)$$

with $w_{a,b,X}$ and φ being real parameters. Once the scattering potential has been characterized using the parameterization given in Equation (18), the matching matrix can be computed by direct exponentiation of the matrix:

(19) $$-i{\left(\hbar v_{F}\right)}^{-1}{\hat{\sigma }}_{x}ℬ=-i\left(\begin{array}{cc} w_{X}e^{i\varphi } & w_{b}\\ \;w_{a} & w_{X}e^{-i\varphi } \end{array}\right)\equiv A$$

Hereafter, we provide explicit examples of the implementation of this procedure and its outcomes.

**Case 1:** $w_{a}=\;w_{b}=\Lambda$ **and** $w_{X}=0$

This case corresponds to consider an interface potential of the form:

(20) $$U\left(x\right)=\hbar v_{F}\left(\begin{array}{cc} \Lambda & 0\\ 0 & \Lambda \end{array}\right)\delta \left(x\right)$$

Following the procedure described before, we obtain the matching matrix:

(21) $$ℛ=\left(\begin{array}{cc} \cos\left(\Lambda \right) & -i\sin\left(\Lambda \right)\\ -i\sin\left(\Lambda \right) & \cos\left(\Lambda \right) \end{array}\right)$$

which is an SU(2) matrix (i.e., det(ℛ)=1 and ${ℛ}^{+}ℛ=1$) parameterized by the dimensionless quantity Λ. The comparison of Equation (21) with Equation (13) shows that the identification ℛ=ℳ is possible by taking −Λ=λ and θ=γ=0. The above comparison makes a link between the dimensionless potential amplitude Λ in Equation (20) and the parameter λ, which is the free parameter coming from the algebraic analysis carried out in Section 2.4. In this way, the physical meaning of the parameter λ can be recognized.

**Case 2:** $w_{a}=\Lambda$ **,** $w_{b}=-\Lambda$ **and** $w_{X}=0$ **(mass term)**

This case corresponds to consider a mass term potential of the form:

(22) $$U\left(x\right)=\hbar v_{F}\left(\begin{array}{cc} \Lambda & 0\\ 0 & -\Lambda \end{array}\right)\delta \left(x\right)$$

The matching matrix corresponding to this interface potential takes the form:

(23) $$ℛ=\left(\begin{array}{cc} \cosh\left(\Lambda \right) & i\sinh\left(\Lambda \right)\\ -i\sinh\left(\Lambda \right) & \cosh\left(\Lambda \right) \end{array}\right)\;$$

which is not unitary (${ℛ}^{+}ℛ\neq 1$) and belongs to the SL(2,C), implying that det(ℛ)=1. It is easy to prove that Equation (23) can be obtained by properly fixing the free parameters that appear in Equation (14).

**Case 3:** $w_{a}=\;w_{b}=0$ **and** $w_{X}=\Lambda$

This case corresponds to consider an interface potential of the form:

(24) $$U\left(x\right)=\hbar v_{F}\left(\begin{array}{cc} 0 & \Lambda e^{-i\varphi }\\ \Lambda e^{i\varphi } & 0 \end{array}\right)\delta \left(x\right)$$

The physical meaning of this kind of interface potential is explained in Appendix A; here, we just comment that the introduction of an off-diagonal potential in the Dirac equation produces a displacement of the K, K’ points in momentum space, with the latter effect being usually the result of lattice deformations, e.g., induced by mechanical strain. The matching matrix corresponding to the interface potential given in Equation (24) takes the form:

(25) $$ℛ=e^{-i\;\Lambda \cos\left(\varphi \right)}\left(\begin{array}{cc} e^{\Lambda \sin\left(\varphi \right)} & 0\\ 0 & e^{-\Lambda \sin\left(\varphi \right)} \end{array}\right)\;$$

which is defined by a pure phase factor $e^{-i\;\Lambda \cos\left(\varphi \right)}$ multiplied by a determinant one-diagonal matrix belonging to SL(2,C). According to our previous discussion, this kind of matching matrices preserves the current density at the interface; thus, they are admissible matching matrices despite the determinant being different from one. A direct proof of this statement is provided here for the sake of completeness. The Jacobi formula for a generic complex coefficient square matrix C implies that det(exp(C))=exp(Tr(C)), with Tr(C) being the trace of C. When C is substituted with A in Equation (19), we get $det\left(ℳ\right)=e^{-2iw_{X}\;cos\left(\varphi \right)}$, with $ℳ=e^{A}$ being the matching matrix of the problem. In this way, we have proven that the determinant of a matching matrix is fixed to one only for diagonal interface potentials, while it is a pure phase factor when off-diagonal terms affect the structure of the interface potential (i.e., when $w_{X}\neq 0$).

## 3. Grain Boundary Hamiltonian Model with Position-Dependent Rotation Angle θ(x)

Before characterizing the scattering properties of a grain boundary, we would like to understand if a phase gradient alone can generate a non-vanishing scattering potential at the grain boundary junction. To answer this question, we need a convenient model in which the phase profile at the interface is a smooth function of the position. Thus, a regularization of the Hamiltonian model presented in Equation (11) is needed. A regularized Hamiltonian model is obtained by taking the rotation angle θ as a position-dependent function θ(x) defining a smeared step function; it provides a gentle matching between the lattice rotation angle $\theta_{L}$ on the left of the junction and $\theta_{R}$, characterizing the right side. In this way, a finite phase gradient is present inside the grain boundary region (i.e., the region close to x=0). The resulting Hamiltonian model cannot be obtained just by making the substitution θ→θ(x) in Equation (4). This is because, according to quantum mechanics prescriptions, terms such as $e^{i\theta \left(x\right)}{\hat{p}}_{x}$ have to be symmetrized to avoid non-Hermitian contributions to the Hamiltonian. After the symmetrization procedure has been implemented, the following regularized grain boundary Hamiltonian is obtained:

(26) $$H_{\theta \left(x\right)}=v_{F}\left(\begin{array}{cc} 0 & \frac{1}{2}\left\{e^{i\theta \left(x\right)},{\hat{p}}_{x}\right\}-e^{i\theta \left(x\right)}i{\hat{p}}_{y}\\ \frac{1}{2}\left\{e^{-i\theta \left(x\right)},{\hat{p}}_{x}\right\}+e^{-i\theta \left(x\right)}i{\hat{p}}_{y} & 0 \end{array}\right),$$

where we have introduced the anticommutator $\left\{\hat{A},\hat{B}\right\}=\hat{A}\hat{B}+\hat{B}\hat{A}$ between generic quantum operators $\hat{A}$ and $\hat{B}$. It is easy to demonstrate that the dependence on θ(x) in Equation (26) can be eliminated using the local unitary transformation:

(27) $$U\left(x\right)=\left(\begin{array}{cc} e^{i\theta \left(x\right)/2} & 0\\ 0 & e^{-i\theta \left(x\right)/2} \end{array}\right).$$

In particular, as anticipated in Section 2.5, we obtain $U^{+}\left(x\right)H_{\theta \left(x\right)}U\left(x\right)=H_{\theta \left(x\right)=0}$, with the latter result being independent of the specific functional form of θ(x). Moreover, terms in the Hamiltonian Equation (26) depending on the phase gradient $\partial_{x}\theta \left(x\right)$ are exactly cancelled by the unitary transformation. According to these arguments, the mismatch of the crystallographic axes at the grain boundary of a Dirac material does not provide disturbance for particles transmission, at least in the framework of the present model. This conclusion crucially depends on the first-order nature of the differential operator representing the kinetic energy of the Dirac particles.

When the Hamiltonian presented in Equation (26) includes a generic interface potential of the form:

(28) $$W=\hbar v_{F}\left(\begin{array}{cc} w_{a} & w_{X}\\ w_{X} & w_{b} \end{array}\right)\delta \left(x\right)\;$$

the complete Hamiltonian takes the form $H_{\theta \left(x\right)}+W$. The application of the unitary transformation in Equation (27) now leads to the transformed Hamiltonian problem $U^{+}\left(x\right)\left(H_{\theta \left(x\right)}+W\right)U\left(x\right)=H_{\theta \left(x\right)=0}+\tilde{W}$, with the transformed interface potential given by:

(29) $$\;\tilde{W}=\hbar v_{F}\left(\begin{array}{cc} w_{a} & w_{X}e^{-i\theta \left(0\right)}\\ w_{X}e^{i\theta \left(0\right)} & w_{b} \end{array}\right)\delta \left(x\right)\;$$

with $w_{a}$, $w_{b}$, and $w_{X}$ being dimensionless real parameters. The transformed potential $\tilde{W}$ explicitly contains a dependence on the phase value θ(0) being assumed at the grain boundary position x=0. This behavior is specific to the Dirac Delta potential, which is appropriate to describe the grain boundary interface in the long wavelength limit. Here, we notice that the relation between θ(0) and $\theta_{R,L}$ depends on the details of the θ(x) dependence. To be specific, a simple model of θ(x) can be expressed, e.g., by the following piecewise function:

(30) $$\theta \left(x\right)=\{x\left(\theta_{R}-\theta_{L}\right)/2+\begin{array}{cc} \theta_{R} & x>d/2\\ \left(\theta_{L}+\theta_{R}\right)/2 & x\in \left[-d/2,d/2\right]\\ \theta_{L} & x<-d/2 \end{array}\;$$

according to which $\theta \left(0\right)=\left(\theta_{L}+\theta_{R}\right)/2$. In Equation (30), where the limit d→0 has to be meant, $x\in \left[-\frac{d}{2},\frac{d}{2}\right]$ represents the grain boundary region. As a final comment, we argue that the scattering problem related to the transformed Hamiltonian $H_{\theta \left(x\right)=0}+\tilde{W}$ can be treated using ordinary boundary conditions, as described in Section 2.5.

## 4. Results of the Scattering Theory

### 4.1. Transmission Properties of a n/n’ Grain Boundary Junction with Unconventional Boundary Conditions

The purpose of this section is to provide simple examples of the use of unconventional matching matrices, and create a link between the transmittance properties of the problem and the specific matching matrix that is adopted. A complete analysis of all of the conduction regimes of the junction (e.g., the p/n case) is beyond the scopes of this section. Thus, we study the scattering problem of a n/n’ grain boundary junction described by the Hamiltonian Equation (6). The peculiar properties of the transmittance are derived using matching matrices with a simplified structure compared to those described in Section 2.4.

The scattering states in the left and right part of the junction are expanded in terms of local eigenstates, taking into account the translational invariance of the scattering region along the y-direction. According to this procedure, the scattering states, which are normalized by the absolute value of the group velocity $v_{x}^{L,R}$ along the propagation direction, can be written in the following general form:

(31) $$\begin{array}{c} \Psi_{L}\left(x,y\right)=e^{ik_{y}y}\left\{\left(\begin{array}{c} 1\\ e^{i\phi } \end{array}\right)\frac{e^{ik_{x}x}}{\sqrt{2v_{x}^{L}}}+ℛ\left(\begin{array}{c} 1\\ -e^{-i\phi } \end{array}\right)\frac{e^{-ik_{x}x}}{\sqrt{2v_{x}^{L}}}\right\}\\ \Psi_{R}\left(x,y\right)=T\;e^{iq_{y}y}\left(\begin{array}{c} 1\\ e^{i\left(\phi_{t}-\theta \right)} \end{array}\right)\frac{e^{iq_{x}x}}{\sqrt{2v_{x}^{R}}} \end{array}.$$

**Figure 2:**
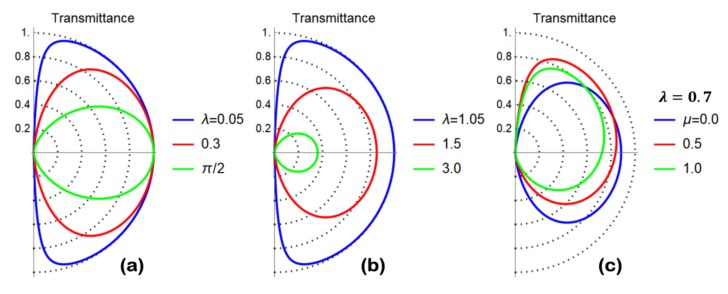
(**a**) Polar plot of the transmittance (transmission probability) ${\left|T\left(\phi \right)\right|}^{2}$ versus the incidence angle ϕ computed according to Equation (32) and setting the model parameters as specified in the legend. The matching matrix that is used belongs to SU(2), and accordingly, full transmission is observed for ϕ=0 incidence, with the latter result being insensitive to the parameter choice and reminiscent of the Klein tunneling phenomenon. (**b**) Polar plot of the transmittance (transmission probability) ${\left|T\left(\phi \right)\right|}^{2}$ versus the incidence angle ϕ computed according to Equation (36) and setting the model parameters as specified in the legend. Diagonal SL(2,C) matching matrix (see Equation (35)) has been used. A reduction of the transmittance is observed for arbitrary values of the incidence angle as the parameter λ is increased. (**c**) Polar plot of the transmittance (transmission probability) ${\left|T\left(\phi \right)\right|}^{2}$ versus the incidence angle ϕ computed according to Equation (38) and setting the model parameters as specified in the legend. A non-diagonal SL(2,C) matching matrix (see Equation (37)) has been used. An anisotropic reduction of the transmittance is observed for the arbitrary values of the incidence angle as the parameter μ is increased. Preferential transport directions are obtained.

Here, $\Psi_{L}\left(x,y\right)$ describes the reflection of a particle belonging to the conduction band (E>0) impinging from the left on the grain boundary interface with a group velocity of $v_{x}^{L}=v_{F}\cos\phi$ and the linear momentum $k_{x}=\frac{E}{\hbar v_{F}}\cos\phi$, ϕ∈[−π/2,π/2], in which the incidence angle is formed by the particle trajectory with the x-axis. The expression of $v_{x}^{L}$ is related to the dispersion relation on the left side of the junction, namely $E=\hbar v_{F}\sqrt{k_{x}^{2}+k_{y}^{2}}$, by the usual relation $v_{x}^{L}=\hbar^{-1}\partial_{k_{x}}E$. In writing the reflected wave contribution (weighted by the reflection coefficient ℛ), we have explicitly taken into account that the reflection angle $\phi_{r}$ is related to the incidence angle ϕ by the relation $\phi_{r}=\pi -\phi$. In the right part of the junction (x>0), the wave function $\Psi_{R}\left(x,y\right)$ describes a transmitted electron with group velocity $v_{x}^{R}=v_{F}\cos\phi_{t}$ and linear momentum $q_{x}=\frac{E}{\hbar v_{F}}\cos\phi_{t}$. Since we are assuming the alignment of the Dirac points of the left and right part of the junction, the dispersion relation of the transmitted electron is given by $E=\hbar v_{F}\sqrt{q_{x}^{2}+q_{y}^{2}}$. Here, it is worth mentioning that the spinor of the transmitted particle explicitly depends on the rotation angle θ characterizing the rotation of the cristallographic axes of the right part of the junction. In the elastic scattering of a translational invariant system along the y-direction, both the energy E and the linear momentum along the y-direction are conserved quantities. This observation implies that $k_{y}=q_{y}$. Observing that $k_{y}=\frac{E}{\hbar v_{F}}\sin\phi$ and $q_{y}=\frac{E}{\hbar v_{F}}\sin\phi_{t}$, we conclude that $\phi =\phi_{t}$. The scattering states in Equation (31) are used to derive the scattering problem equation $\Psi_{R}\left(x=0,y\right)=ℳ\Psi_{L}\left(x=0,y\right)$, whose structure is fixed once the matching matrix ℳ has been specified. Once the scattering equation has been solved, the scattering coefficients ℛ and T are determined; moreover, current density conservation implies ${\left|ℛ\right|}^{2}+{\left|T\right|}^{2}=1$. The transmittance ${\left|T\right|}^{2}$ is an angle-resolved quantity that is directly related to the scattering properties of the interface and determines the differential conductance of the system. In the following, several relevant cases are treated, while the transmittance curves as a function of the incidence angle ϕ are presented in Figure 2a–c.

**Case 1: SU(2) matching matrix (Equation (13))**

We have solved the scattering problem using the SU(2) matching matrix given in Equation (13) by fixing γ=0. The transmittance of the problem has been derived and takes the following (energy-independent) form:

(32) $${\left|T\left(\phi \right)\right|}^{2}=\frac{1}{\cos^{2}\left(\lambda \right)+\sin^{2}\left(\lambda \right)\sec^{2}\left(\phi \right)}\;$$

The main property of Equation (32) is that ${\left|T\left(\phi =0\right)\right|}^{2}=1$ for any choice of the parameter λ (see Figure 2a). This behavior, which is reminiscent of the Klein tunneling, is clearly inappropriate to describe the grain boundary physics for which a reduction of the transmittance is expected when the strength of the scattering potential (controlled by λ) is increased. No dependence on θ is present in the transmittance, since the transmission coefficient T(ϕ) presents an irrelevant prefactor $e^{i\theta /2}$.

**Case 2: Matching matrix belonging to** SL(2,C) **; *example 1***

We have solved the scattering problem using the SL(2,C) matching matrix:

(33) $$ℳ=\left(\begin{array}{cc} \sqrt{1+\lambda^{2}}\;e^{i\theta /2} & -i\lambda \;e^{i\theta /2}\\ \;i\lambda \;e^{-i\theta /2} & \sqrt{1+\lambda^{2}}\;e^{-i\theta /2} \end{array}\right),$$

which is a special case of the matching matrix given in Equation (14), and it is appropriate to describe the interface potentials proportional to ${\hat{\sigma }}_{z}$ (mass term). The solution of the scattering problem provides the scattering coefficients:

(34) $$\begin{array}{c} T\left(\phi \right)=\frac{e^{i\theta /2}}{\sqrt{1+\lambda^{2}}}\\ ℛ\left(\phi \right)=\frac{i\lambda e^{i\phi }}{\sqrt{1+\lambda^{2}}} \end{array},$$

implying an angle-independent transmittance given by ${\left|T\left(\phi \right)\right|}^{2}=1/\left(1+\lambda^{2}\right)$, which is a decreasing function of the scattering strength parameter λ. This kind of matching matrix seems to be appropriate to describe the conductance reduction induced by a grain boundary region, even though no dependence on θ is detected. The above findings confirm the confining properties of a potential proportional to ${\hat{\sigma }}_{z}$.

**Case 3: Matching matrix belonging to** SL(2,C) **; *example 2***

We have solved the scattering problem using the diagonal SL(2,C) matching matrix:

(35) $$ℳ=\left(\begin{array}{cc} \lambda \;e^{i\theta /2} & 0\\ 0 & \lambda^{-1}\;e^{-i\theta /2} \end{array}\right).$$

The transmittance of the problem has been derived, and takes the following (energy-independent) form:

(36) $$\;{\left|T\left(\phi \right)\right|}^{2}=\frac{4\lambda^{2}\cos^{2}\left(\phi \right)}{1+\lambda^{4}+2\lambda^{2}\cos\left(2\phi \right)}\;$$

A reduction of the transmittance (see Figure 2b) is observed when the scattering strength parameter λ is increased, with the latter behavior being appropriate to describe the grain boundary physics.

**Case 4: Matching matrix belonging to** SL(2,C) **; *example 3***

We have solved the scattering problem using the following SL(2,C) matching matrix:

(37) $$ℳ=\left(\begin{array}{cc} \lambda \;e^{i\theta /2} & -i\mu \;e^{i\theta /2}\\ 0 & \lambda^{-1}\;e^{-i\theta /2} \end{array}\right),$$

which depends on the dimensionless parameters λ and μ. The transmittance of the problem has been derived, and takes the following (energy-independent) form:

(38) $${\left|T\left(\phi \right)\right|}^{2}=\frac{4\lambda^{2}\cos^{2}\left(\phi \right)}{1+\lambda^{4}+\lambda^{2}\mu^{2}+2\lambda \left[\lambda \cos\left(2\phi \right)+\mu \left(\lambda^{2}-1\right)\sin\phi \right]}\;$$

A reduction of the transmittance is observed when the scattering strength parameter λ is increased, with the latter behavior being appropriate to describe the grain boundary physics. Moreover, depending on the choice of parameters, preferential transport directions are obtained (see Figure 2c).

The analysis of cases 1–4 shows that the grain boundary transmittance can be independent of θ, despite the matching matrix and the spinorial wave function (see Equation (31)) presenting a dependence on this parameter. Furthermore, direct computation (not reported here) shows that θ-independent transmittance is not a peculiarity of the single barrier case, but rather survives in the double barrier case even when the different regions of the conduction channel are described by a Dirac Hamiltonian rotated by different angles. The above behavior suggests that the algebraic deduction of the matching matrix structure (see Section 2.4) is not able to capture the hidden dependence on the rotation angle θ, which in principle can be important.

To clarify this point, in the subsequent section, we analyze a simple model of the n/n’ grain boundary junction based on the Hamiltonian treatment proposed in Section 3. The physical conditions under which the system presents a θ-dependent differential conductance are studied.

### 4.2. Grain Boundary Junction with θ-Dependent Differential Conductance

We now formulate a theory of the n/n’ grain boundary junction based on the Hamiltonian model presented in Section 3. Let us consider the grain boundary junction described by the Hamiltonian $H=H_{\theta \left(x\right)}+W\left(x\right)+V_{s}\left(x\right)$, where W(x) is the interface potential introduced in Equation (28), while $V_{s}\left(x\right)$ is a step-like potential:

(39) $$V_{s}\left(x\right)=\{\begin{array}{c} \begin{array}{cc} 0 & x<0 \end{array}\\ \begin{array}{cc} V_{0}I_{2\times 2} & x>0 \end{array} \end{array}$$

mimicking a potential profile induced by charge transfer at the grain boundary. Such potential is assumed to be diagonal in the sublattice representation, $I_{2\times 2}=diag\left(1,1\right)$ representing the identity in the sublattice space. Using the unitary transformation in Equation (27), the Hamiltonian problem can be written in the equivalent form $U^{+}\left(x\right)\left(H_{\theta \left(x\right)}+W\left(x\right)+V_{s}\left(x\right)\right)U\left(x\right)=H_{\theta \left(x\right)=0}+\tilde{W}\left(x\right)+V_{s}\left(x\right)\equiv ℋ$, where $\tilde{W}\left(x\right)$ is given in Equation (29), while $V_{s}\left(x\right)$ is invariant under unitary transformation. The junction described by ℋ can be treated using ordinary boundary conditions, which are implemented using the matching matrix formulation given in Section 2.5. Since we are interested in verifying the existence of preferential transport directions, we focus our analysis on the interface potential:

(40) $$\tilde{W}\left(x\right)=\hbar v_{F}\left(\begin{array}{cc} w_{a} & w_{X}e^{-i\theta \left(0\right)}\\ w_{X}e^{i\theta \left(0\right)} & 0 \end{array}\right)\delta \left(x\right),$$

which is obtained from Equation (29) by setting $w_{b}=0$. The matching matrix associated with Equation (40) is given by:

(41) $$ℳ=\left(\begin{array}{cc} e^{-iℊ} & 0\\ \frac{e^{i\theta \left(0\right)}\left(e^{-iℊ}-e^{-i{ℊ}^{*}}\right)\alpha }{e^{2i\theta \left(0\right)}-1} & e^{-i{ℊ}^{*}} \end{array}\right)\;$$

which defines a SL(2,C) boundary condition with parameters $ℊ=w_{X}e^{i\theta \left(0\right)}$, ${ℊ}^{*}=w_{X}e^{-i\theta \left(0\right)}$, and $w_{a}=\alpha w_{X}$. Equation (41) presents an explicit dependence on the rotation angle θ(0) at the grain boundary junction, and thus a dependence of the transport properties of the system on this parameter is expected.

**Figure 3:**
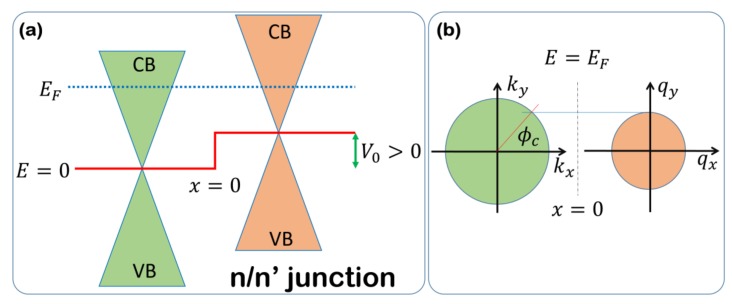
(**a**) Bands alignment of the n/n’ junction model considered in the main text. Grain boundary interface is located in x=0. The system Fermi level is located above the Dirac points, and thus, a n-type conduction regime is established. (**b**) In the translational invariant system along the y-direction, the corresponding linear momentum is a conserved quantum number in the scattering events. When the incidence angle ϕ is greater than the critical angle $\phi_{c}$, the y-component of the linear momentum cannot be conserved, and no current can flow through the interface.

In order to present the results of the model, we focus our attention on the n/n’ junction described in Figure 3a,b. The scattering problem related to the conduction properties of the junction is solved using the scattering states given in Equation (31) with θ=0. However, differently from the case treated in Section 4.1, the dispersion relation in the distinct sides of the junction, namely $E_{k}^{\left(L/R\right)}$, are different, and in particular we have that $E_{k}^{\left(L\right)}=\hbar v_{F}\sqrt{k_{x}^{2}+k_{y}^{2}}=\hbar v_{F}\left|k\right|$ for x<0 and $E_{k}^{\left(R\right)}=\hbar v_{F}\sqrt{q_{x}^{2}+q_{y}^{2}}+V_{0}=\hbar v_{F}\left|q\right|+V_{0}$ for x>0. The group velocities are the same as considered before, and remain unaffected by the presence of a potential step at the interface. Energy E of a particle is conserved during the scattering event, and thus, we can set $E_{k}^{\left(L\right)}=E_{k}^{\left(R\right)}=E$. The latter relation allow us to deduce the moduli of the linear momenta on the different junction sides, namely $\left|k\right|=E/\hbar v_{F}$ and $\left|q\right|=\left(E-V_{0}\right)/\hbar v_{F}$ with $E-V_{0}>0$, since we are considering an n/n’ junction. The y-component of the linear momentum on different sides of the junction is thus given by $k_{y}=\left|k\right|\sin\phi$ and $q_{y}=\left|q\right|\sin\phi_{t}$, while translational invariance implies $k_{y}=q_{y}$. Conservation of the y-component of the linear momentum implies the following relation between the incidence and the transmission angle:

(42) $$\phi_{t}=\sin^{-1}\left(\frac{E}{E-V_{0}}\sin\phi \right).$$

Since the quantity $\frac{E}{E-V_{0}}>1$, there exists a critical angle of incidence for which Equation (42) cannot be satisfied, and no current can flow through the interface. Such a critical value takes the following form:

(43) $$\phi_{c}=\sin^{-1}\left(\frac{E-V_{0}}{E}\right)\;$$

Once the scattering problem has been solved using the matching matrix given in Equation (41), the angle-resolved transmittance ${\left|T\left(\phi \right)\right|}^{2}$ is derived. The zero-temperature differential conductance of the junction $G_{nn^{\prime }}$ is related to the transmittance evaluated at the Fermi level, $E=E_{F}$ by the following relation:

(44) $$G_{nn^{\prime }}=g_{s}g_{v}\frac{e^{2}}{h}\left(\frac{k_{F}ℒ}{2\pi }\right)\int_{-\phi_{c}}^{\phi_{c}}d\phi \;\left[{\left|T\left(\phi \right)\right|}^{2}\;\cos\phi \right],$$

with $g_{s}g_{v}=4$ being a degeneracy factor coming from the spin ($g_{s}=2$) and the valley ($g_{v}=2$) degeneracy, ℒ being the transverse dimension of the junction assumed to be a macroscopic quantity, and $k_{F}=E_{F}/\left(\hbar v_{F}\right)$ being the modulus of the Fermi wave vector. Here is worth mentioning that the Fermi level can be tuned by using a back gate with the twofold effect of modulating the number of transverse channels $N\left(E_{F}\right)=g_{s}g_{v}\left(\frac{k_{F}ℒ}{2\pi }\right)$ involved in the conduction and changing the value of the critical angle $\phi_{c}$, which is an energy-sensitive quantity.

Before treating the general case of $V_{0}\neq 0$, we study the solution of the scattering problem under the simplifying assumption $V_{0}=0$, which allow us to obtain the following simple expression for the angle-resolved transmittance:

(45) $${\left|T\left(\phi \right)\right|}^{2}=\frac{4}{4\cosh^{2}\left(w_{X}\sin\theta \left(0\right)\right)+\sinh^{2}\left(w_{X}\sin\theta \left(0\right)\right){\left[2\tan\phi +\alpha \sec\phi \csc\theta \left(0\right)\right]}^{2}}\;$$

**Figure 4:**
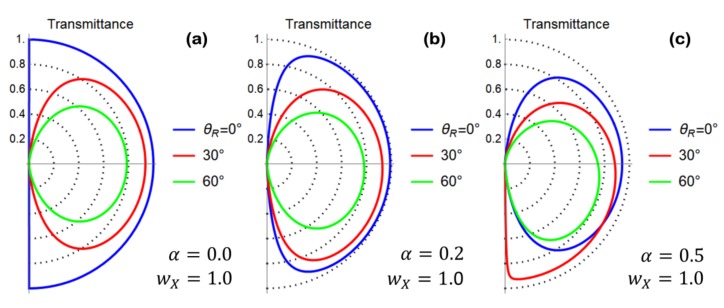
(**a**) Polar plot of the transmittance computed according to Equation (45) by setting the model parameters as shown in the figure legend. Preferential transport directions are absent, while the transmission probability is reduced as the misorientation angle $\theta_{R}$ is increased. (**b**) Polar plot of the transmittance computed according to Equation (45) by setting α=0.2 and $w_{X}=1$. Preferential transport directions are observed. (**c**) Polar plot of the transmittance computed according to Equation (45) by setting α=0.5 and $w_{X}=1$. Preferential transport directions are observed.

A direct inspection of Equation (45) evidences a dependence on θ(0), which is the relic of the cristallographic axes mismatching between the two sides of the grain boundary junction. In the following analysis, we use the model for θ(x) given in Equation (30) with $\theta_{L}=0$. Accordingly, we set $\theta \left(0\right)=\theta_{R}/2$. Furthermore, the analysis of the formation energy of a graphene grain boundary as a function of the misorientation angle $\theta_{R}$ shows an M-shaped behavior with a local minimum at $\theta_{R}≅30{}^{\circ}$ and absolute minima in $\theta_{R}≅0{}^{\circ}$ and $\theta_{R}≅60{}^{\circ}$ [19]. In Figure 4a–c, we have studied the behavior of Equation (45) as a function of the relevant junction parameters. A reduction of the transmission probability is observed as the misorientation angle at the grain boundary $\theta_{R}$ is increased. Preferential transport directions are clearly related to a non-vanishing value of the parameter α.

**Figure 5:**
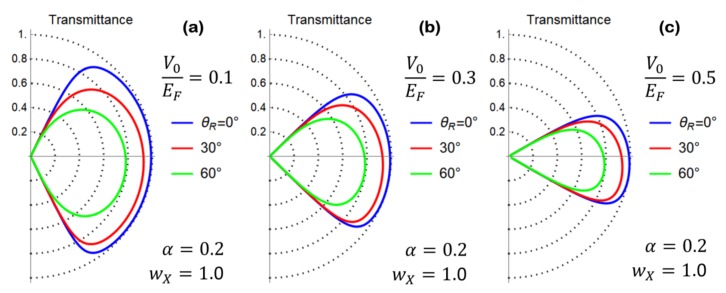
Polar plot of the transmittance of the n/n’ junction computed by setting the interface parameters as α=0.2 and $w_{X}=1.0$. Different curves in each panel refer to different misorientation angles $\theta_{R}$, as indicated in the figure legend. The potential step has been fixed as $\frac{V_{0}}{E_{F}}=0.1,\;0.3,\;0.5$ in panels (**a**–**c**), respectively. Due to the critical angle reduction for increasing values of $V_{0}$, a suppression of the junction transmission at high incidence angles is clearly visible.

The general expression of the transmittance pertaining to the $V_{0}\neq 0$ case is quite lengthy, and thus, we only present a numerical evaluation of it. This analysis is performed in Figure 5a–c, where the effect on the transmittance of a finite potential step at the interface is presented, setting the values of the remaining junction parameters as done in Figure 4b. Here, we observe that the critical angle $\phi_{c}$ of the scattering problem is an energy-sensitive quantity that is reduced when the potential step intensity is increased. Figure 5 clearly shows the effect of the critical angle reduction, and suppression of the junction transmission at high incidence angles is clearly visible.

**Figure 6:**
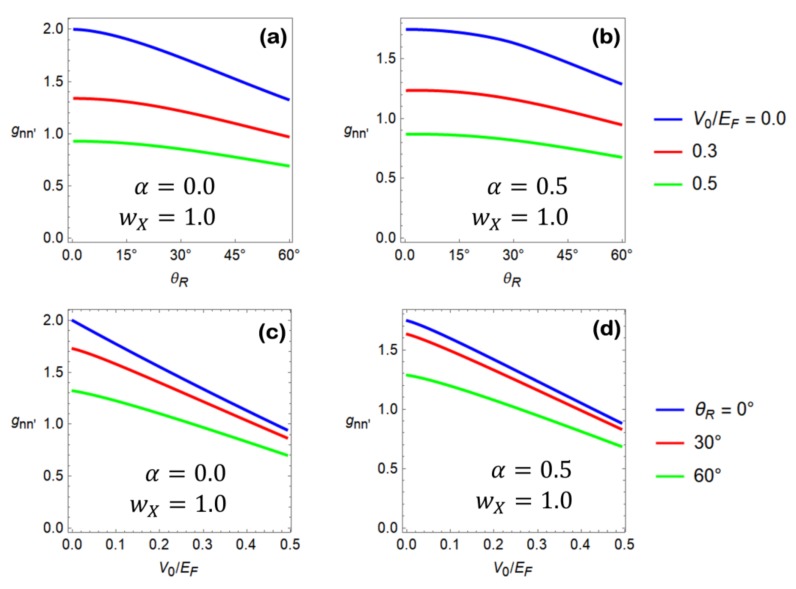
(**a**,**b**) Dimensionless differential conductance $g_{nn^{\prime }}$ as a function of the misorientation angle $\theta_{R}$. Different curves pertain to different values of the intensity of the potential step, as indicated in the figures legend. Matching matrix parameters α=0 and α=0.5 are used in the computation of panel (**a**) and (**b**), respectively. (**c**), (**d**) Dimensionless differential conductance $g_{nn^{\prime }}$ as a function of the potential step intensity $V_{0}$. Different curves pertain to different values of the misorientation angle, as indicated in the figures legend. Panel (**c**) is obtained by fixing α=0, while panel (**d**) is obtained by fixing α=0.5.

In Figure 6a–d, we report the differential conductance given in Equation (44) normalized to the quantity $g_{s}g_{v}\frac{e^{2}}{h}\left(\frac{k_{F}ℒ}{2\pi }\right)\equiv G_{0}$, with the resulting dimensionless quantity being $g_{nn^{\prime }}=G_{nn^{\prime }}/G_{0}$. In particular, in Figure 6a,b, we show $g_{nn^{\prime }}$ as a function of the misorientation angle $\theta_{R}$ for different values of the potential step and of the matching matrix parameters. A suppression of the junction conductance as a function of $\theta_{R}$ is observed, in which the latter behavior is more pronounced for small values of the potential step intensity.

In Figure 6c,d we show $g_{nn\prime }$ as a function of the potential step intensity $V_{0}$. Different curves are related with distinct choices of the misoriantation angle of the grain boundary junction. An almost linear lowering of the $g_{nn^{\prime }}$ versus $V_{0}$ curves is obtained, which is related to the critical angle reduction causing the progressive closure of the conduction channels.

The general conclusion associated with the analysis performed in Figure 6 is that grain boundary junctions with large misorientation angles manifest the tendency to be more opaque compared to those with a better lattice matching. Moreover, charge transfer at the grain boundary interface induces a potential step whose effect is relevant in determining the transparency of the interface.

## 5. Conclusions

We have proposed a continuous model to study the grain boundary effects in graphene. The model provides a description of the grain boundary based on a Dirac Hamiltonian written in a rotated side-dependent reference frame describing crystallographic axes mismatching at a grain boundary junction. We have shown that the scattering problem related to the transmission properties of a grain boundary junction requires modified boundary conditions, which can be implemented by using the matching matrix method in the scattering problem. We have characterized the algebraic properties of all of the possible matching matrices showing that the SU(2) and SL(2,C) matching matrices are admissible. In particular, we have proven that the SU(2) matching matrices support Klein tunneling, and are not adequate in describing the conductance lowering that is associated with a grain boundary physics, which is instead captured by SL(2,C) matching matrices. We have studied specific matching matrix examples and the associated transmittance properties. It has been demonstrated that under opportune assumptions, preferential transport directions are supported by the interface microscopic properties, which are encoded in our approach by the matching matrix structure.

Moreover, using a space-dependent unitary transformation, we have formulated a grain boundary model where usual boundary conditions can be used. Conditions to observe scattering properties related to the crystallographic axes mismatching at the grain boundary interface are studied. Reduction of the junction conductance has been demonstrated as the effect of the misorientation angle between the two junction sides.

Compared to alternative theoretical methods [27,28,29,30], the present approach does not require a tight-binding model of the linear defect (grain boundary) and boundary conditions, which are expressed via the matching matrix method, are derived using the algebraic properties of the scattering problem.

The proposed theory provides a phenomenological model to study grain boundary physics within the scattering approach, and represents *per se* an important enrichment of the scattering theory of graphene, having the potential to stimulate new experiments and further theoretical investigations. 

## Appendix A

Let us consider the rotated Dirac Hamiltonian presented in (4) complemented by the off-diagonal potential in the sublattice space $\hat{V}=V{\hat{\sigma }}_{x}$. The resulting Hamiltonian in momentum space takes the form

(A1) $$H\left(k\right)=H_{\theta }\left(k\right)+\hat{V}=\hbar v_{F}\left(\begin{array}{cc} 0 & e^{i\theta }\left(k_{x}-ik_{y}\right)\\ e^{-i\theta }\left(k_{x}+ik_{y}\right) & 0 \end{array}\right)+\left(\begin{array}{cc} 0 & V\\ V & 0 \end{array}\right).$$

The spectrum of the problem is obtained by solving the equation det[H(k)−1E]=0 with respect to E. Straightforward computation shows that the dispersion relations of the conduction and valence bands take the following form:

(A2) $$E_{\pm }=\pm \hbar v_{F}\sqrt{{\left(k_{x}+\frac{V\cos\theta }{\hbar v_{F}}\right)}^{2}+{\left(k_{y}+\frac{V\sin\theta }{\hbar v_{F}}\right)}^{2}},$$

which clearly shows a dependence on the rotation angle θ which is absent when the off-diagonal potential is switched off (V=0). Moreover, the Dirac point, which is defined as the point in the momentum space where $E_{+}=E_{-}$, is displaced from the original K point in momentum space by the vector $\delta K=-\left(\frac{V\cos\theta }{\hbar v_{F}},\frac{V\sin\theta }{\hbar v_{F}}\right)$. In real systems, Dirac cone displacement from the unperturbed K point is related to strain-induced change in the graphene electronic structure [34]. Since grain boundary regions present defective lattice accompanied by mechanical deformations, the off-diagonal potential introduced in (A1) can well emulate strain-induced modifications of the electronic properties. Clearly, in modeling these effects non-vanishing off-diagonal potential is only admissible in close vicinity of the grain boundary region, while it is expected to be irrelevant when an ordered (without defects and vacancies) graphene lattice is restored. The dependence of the spectrum in (A2) on the rotation angle θ is induced by the properties of the Hamiltonian under unitary transformations. Indeed, rotated Hamiltonian $H_{\theta }\left(k\right)$ is unitarily equivalent to the ordinary graphene Hamiltonian according to the relation $U^{+}H_{\theta }\left(k\right)U=H_{\theta =0}\left(k\right)$ with

(A3) $$U=\left(\begin{array}{cc} e^{i\theta /2} & 0\\ 0 & e^{-i\theta /2} \end{array}\right).$$

Accordingly, the spectrum of $H_{\theta }\left(k\right)$ does not depend on θ. The same conclusion is valid in the presence of a diagonal potential. To demonstrate this point, let us apply the same argument to the Hamiltonian $H=H_{\theta }\left(k\right)+W$, with

(A4) $$W=\left(\begin{array}{cc} V_{A} & 0\\ 0 & V_{B} \end{array}\right).$$

We observe that the transformed Hamiltonian takes the form $U^{+}HU=H_{\theta =0}\left(k\right)+W$ since W is invariant under unitary transformation, i.e., $U^{+}WU=W$. The above observation implies that the energy spectrum remains insensitive to the rotation angle even in the presence of a diagonal single-particle potential W.

On the other hand, when the above procedure is applied to the Hamiltonian $H\left(k\right)=H_{\theta }\left(k\right)+\hat{V}$ considered in (A1), we obtain $U^{+}H\left(k\right)U=H_{\theta =0}\left(k\right)+U^{+}\hat{V}U$ with

(A5) $$U^{+}\hat{V}U\;=\left(\begin{array}{cc} 0 & Ve^{-i\theta }\\ Ve^{i\theta } & 0 \end{array}\right),$$

the latter being the transformed potential. Since $U^{+}\hat{V}U$ retains a dependence on the rotation angle, a θ-dependent energy spectrum is obtained for the Hamiltonian in (A1). The above results suggest that the relative angle formed by the crystallographic axes of the two sides of a grain boundary junction can affect the scattering properties of the system in the presence of an off-diagonal potential in the sublattice indices. This conclusion is clearly supported by the arguments presented in Section 4.2.

## References

[1] Das Sarma S., Adam S., Hwang E.H., Rossi E. (2011). Electronic transport in two-dimensional graphene. Rev. Mod. Phys..

[2] Allain P.E., Fuchs J.N. (2011). Klein tunneling in graphene: Optics with massless electrons. Eur. Phys. J. B.

[3] Rusin T.M., Zawadzki W. (2008). Zitterbewegung of electrons in graphene in a magnetic field. Phys. Rev. B.

[4] Tikhonenko F.V., Kozikov A.A., Savchenko A.K., Gorbachev R.V. (2009). Transition between Electron Localization and Antilocalization in Graphene. Phys. Rev. Lett..

[5] Ostrovsky P.M., Gornyi I.V., Mirlin A.D. (2008). Theory of anomalous quantum Hall effects in graphene. Phys. Rev. B.

[6] Cheianov V.V., Fal’ko V., Altshuler B.L. (2007). The Focusing of Electron Flow and a Veselago Lens in Graphene p-n Junctions. Science.

[7] Banszerus L., Schmitz M., Engels S., Dauber J., Oellers M., Haupt F., Watanabe K., Taniguchi T., Beschoten B., Stampfer C. (2015). Ultrahigh-mobility graphene devices from chemical vapor deposition on reusable copper. Sci. Adv..

[8] Papageorgiou D.G., Kinloch I.A., Young R.J. (2017). Mechanical properties of graphene and graphene-based nanocomposites. Prog. Mater. Sci..

[9] Lee S.-M., Kim J.-H., Ahn J.-H. (2015). Graphene as a flexible electronic material: Mechanical limitations by defect formation and efforts to overcome. Mater. Today.

[10] Lemme M.C., Echtermeyer T.J., Baus M., Kurz H. (2007). A Graphene Field-Effect Device. IEEE Electron Device Lett..

[11] Di Bartolomeo A., Santandrea S., Giubileo F., Romeo F., Petrosino M., Citro R., Barbara P., Lupina G., Schroeder T., Rubino A. (2013). Effect of back-gate on contact resistance and on channel conductance in graphene-based field-effect transistors. Diam. Relat. Mater..

[12] Giubileo F., Di Bartolomeo A. (2017). The role of contact resistance in graphene field-effect devices. Prog. Surf. Sci..

[13] Di Bartolomeo A., Giubileo F., Iemmo L., Romeo F., Santandrea S., Gambardella U. (2013). Transfer characteristics and contact resistance in Ni- and Ti-contacted graphene-based field-effect transistors. J. Phys. Condens. Matt..

[14] Giubileo F., Di Bartolomeo A., Martucciello N., Romeo F., Iemmo L., Romano P., Passacantando M. (2016). Contact Resistance and Channel Conductance of Graphene Field-Effect Transistors under Low-Energy Electron Irradiation. Nanomaterials.

[15] Di Bartolomeo A., Giubileo F., Romeo F., Sabatino P., Carapella G., Iemmo L., Schroeder T., Lupina G. (2015). Graphene field effect transistors with niobium contacts and asymmetric transfer characteristics. Nanotechnology.

[16] Di Bartolomeo A., Giubileo F., Iemmo L., Romeo F., Russo S., Unal S., Passacantando M., Grossi V., Cucolo A.M. (2016). Leakage and field emission in side-gate graphene field effect transistors. Appl. Phys. Lett..

[17] Biró L.P., Lambin P. (2013). Grain boundaries in graphene grown by chemical vapor deposition. New J. Phys..

[18] Jauregui L.A., Cao H., Wu W., Yu Q., Chen Y.P. (2011). Electronic properties of grains and grain boundaries in graphene grown by chemical vapor deposition. Solid State Commun..

[19] Zhang X., Xu Z., Yuan Q., Xin J., Ding F. (2015). The favourable large misorientation angle grain boundaries in graphene. Nanoscale.

[20] Huang P.Y., Ruiz-Vargas C.S., van der Zande A.M., Whitney W.S., Levendorf M.P., Kevek J.W., Garg S., Alden J.S., Hustedt C.J., Zhu Y., Park J., McEuen P.L., Muller D.A. (2011). Grains and grain boundaries in single-layer graphene atomic patchwork quilts. Nature.

[21] Yazyev O.V., Chen Y.P. (2014). Polycrystalline graphene and other two-dimensional materials. Nat. Nanotechnol..

[22] Tsen A.W., Brown L., Levendorf M.P., Ghahari F., Huang P.Y., Havener R.W., Ruiz-Vargas C.S., Muller D.A., Kim P., Park J. (2012). Tailoring Electrical Transport Across Grain Boundaries in Polycrystalline Graphene. Science.

[23] Cummings A.W., Duong D.L., Nguyen V.L., Van Tuan D., Kotakoski J., Barrios Vargas J.E., Lee Y.H., Roche S. (2014). Charge Transport in Polycrystalline Graphene: Challenges and Opportunities. Adv. Mater..

[24] Lahiri J., Lin Y., Bozkurt P., Oleynik I.I., Batzill M. (2010). An extended defect in graphene as a metallic wire. Nat. Nanotechnol..

[25] Ferreira A., Xu X., Tan C.-L., Bae S.-K., Peres N.M.R., Hong B.-H., Özyilmaz B., Castro Neto A.H. (2011). Transport properties of graphene with one-dimensional charge defects. Europhys. Lett..

[26] Radchenko T.M., Shylau A.A., Zozoulenko I.V., Ferreira A. (2013). Effect of charged line defects on conductivity in graphene: Numerical Kubo and analytical Boltzmann approaches. Phys. Rev. B.

[27] Rodrigues J.N.B., Peres N.M.R., Lopes dos Santos J.M.B. (2012). Scattering by linear defects in graphene: A continuum approach. Phys. Rev. B.

[28] Rodrigues J.N.B., Peres N.M.R., Lopes dos Santos J.M.B. (2013). Scattering by linear defects in graphene: A tight-binding approach. J. Phys. Condens. Matt..

[29] Páez C.J., Pereira A.L.C., Rodrigues J.N.B., Peres N.M.R. (2015). Electronic transport across linear defects in graphene. Phys. Rev. B.

[30] Rodrigues J.N.B. (2016). Intervalley scattering of graphene massless Dirac fermions at 3-periodic grain boundaries. Phys. Rev. B.

[31] Foà Torres L.E.F., Roche S., Charlier J.C. (2014). Introduction to Graphene-Based Nanomaterials: From Electronic Structure to Quantum Transport.

[32] Fleet L. (2015). Jump across: Valleytronics. Nature Phys..

[33] McKellar B.H.J., Stephenson G.J. (1987). Relativistic quarks in one-dimensional periodic structures. Phys. Rev. C.

[34] Huang M., Yan H., Heinz T.F., Hone J. (2010). Probing Strain-Induced Electronic Structure Change in Graphene by Raman Spectroscopy. Nano Lett..

